# The application of blood flow sound contrastive learning to predict arteriovenous graft stenosis of patients with hemodialysis

**DOI:** 10.1371/journal.pone.0308385

**Published:** 2024-08-16

**Authors:** Hugo Y.-H. Lin, Tiffany Shien, Juan-Wei Xu, Yen-Jung Kuo, Phang-Lang Chen, Sheng-Wen Niu, I-Ching Kuo, Hsuan-Fu Kuo, Kai-Chieh Yang, Yi-Ren Yeh

**Affiliations:** 1 Department of Internal Medicine, Kaohsiung Municipal Ta-Tung Hospital, Kaohsiung Medical University, Kaohsiung, Taiwan; 2 Division of Nephrology, Kaohsiung Medical University Hospital, Kaohsiung, Taiwan; 3 Department of Medicine, College of Medicine, Kaohsiung Medical University, Kaohsiung, Taiwan; 4 Geisel School of Medicine at Dartmouth, Hanover, NH, United States of America; 5 Department of Mathematics, National Kaohsiung Normal University, Kaohsiung, Taiwan; 6 Department of Post Baccalaureat Medicine, College of Medicine, Kaohsiung Medical University, Kaohsiung, Taiwan; 7 Department of Biological Chemistry, School of Medicine, University of California, Irvine, CA, United States of America; 8 Division of Cardiology, Kaohsiung Medical University Hospital, Kaohsiung, Taiwan; 9 KURA CARE INC, San Diego, CA, United States of America; Jordan University of Science and Technology Faculty of Computer and Information Technology, JORDAN

## Abstract

End-stage kidney disease (ESKD) presents a significant public health challenge, with hemodialysis (HD) remaining one of the most prevalent kidney replacement therapies. Ensuring the longevity and functionality of arteriovenous accesses is challenging for HD patients. Blood flow sound, which contains valuable information, has often been neglected in the past. However, machine learning offers a new approach, leveraging data non-invasively and learning autonomously to match the experience of healthcare professionas. This study aimed to devise a model for detecting arteriovenous grafts (AVGs) stenosis. A smartphone stethoscope was used to record the sound of AVG blood flow at the arterial and venous sides, with each recording lasting one minute. The sound recordings were transformed into mel spectrograms, and a 14-layer convolutional neural network (CNN) was employed to detect stenosis. The CNN comprised six convolution blocks with 3x3 kernel mapping, batch normalization, and rectified linear unit activation function. We applied contrastive learning to train the pre-training audio neural networks model with unlabeled data through self-supervised learning, followed by fine-tuning. In total, 27,406 dialysis session blood flow sounds were documented, including 180 stenosis blood flow sounds. Our proposed framework demonstrated a significant improvement (p<0.05) over training from scratch and a popular pre-trained audio neural networks (PANNs) model, achieving an accuracy of 0.9279, precision of 0.8462, and recall of 0.8077, compared to previous values of 0.8649, 0.7391, and 0.6538. This study illustrates how contrastive learning with unlabeled blood flow sound data can enhance convolutional neural networks for detecting AVG stenosis in HD patients.

## 1 Introduction

The prevalence rates of end-stage kidney disease (ESKD) in the United States and Europe are estimated up to 1100 to 2400 per million population [1, 2]. Hemodialysis (HD) is still one of the major kidney replacement therapies (KRTs). The arteriovenous access complications are recognized as a significant contributor to patient morbidity and the financial burden on healthcare payments. The expense of Medicare for dialysis vascular access-related administrations was $2.8 billion in the United States [3]. The payment for the National Health Service in endovascular interventions alone to preserve the patency of vascular access in patients with HD is assessed at £84 million in the United Kingdom [4]. Accordingly, vascular access dysfunction has developed into a worldwide issue. Significant endeavors have been made to lessen the jeopardy of vascular access dysfunction and maintain access patency.

There are several critical requirements for vascular access in HD. Firstly, it must be suitable for repeated circulatory access. Secondly, it should allow for adequate blood flow to enable efficient dialysis. Thirdly, it should be associated with minimal complications [5, 6]. While monitoring intravascular pressure and estimating access blood flow are valuable, they do not eliminate the risk of vascular access dysfunction. Approximately 20-30% of hospital admissions among HD patients are linked to vascular access dysfunction and failure [7]. This dysfunction can stem from various factors, such as the failure of an AVF to mature, the loss of a mature AVF, or the failure of an arteriovenous graft (AVG). The primary pathogenesis of vascular access dysfunction is neointimal hyperplasia, leading to stenosis and thrombosis. Furthermore, central catheter-related infections can contribute to vascular access issues. Given the high prevalence rate of vascular access dysfunction, early detection of malfunction by patients or their families is crucial for timely intervention and prevention of hospital admissions. However, there is currently no ideal early warning system for the stenosis of arteriovenous access in clinical practice, and the evaluation of the arteriovenous access function depends entirely on the medical staff. With the COVID pandemic, telemedicine with remote health care is important for long-term health management [8]. In recent years, the Internet of Things (IoT) architecture application and smart mobile device technology have developed rapidly [9], which will bring new application opportunities to the smart healthcare industry, and further use of the evolutionary optimization algorithm technology of computing intelligence may solve the unmet need for early prevention of AVG stenosis.

Artificial intelligence (AI) solutions exist in all medical and nonmedical fields. New algorithms have advanced to deal with complex clinical circumstances where medical technologies have reached capacity [10]. Medical registries gotten machine learning (ML) resolutions for a superior forecast of occasions that beat human precision [11]. In our work, we are focused on collecting and analyzing blood flow sound data to detect arteriovenous graft failure using deep learning methods [12]. If the failure of an AVG could be early detected through blood flow sound recordings, this would fill this gap by providing a quick, non-invasive, and cost-effective objective measure. Early detection of this failure is crucial for timely intervention and can improve patient outcomes. By applying deep learning techniques to this data, we aim to improve the accuracy and efficiency of diagnosis, ultimately leading to better patient care.

One challenge in applying deep learning for medical diagnosis is the lack of sufficient data, particularly when it comes to collecting and labeling specialized data such as blood flow sounds. This is the case for our work on detecting arteriovenous graft failure using blood flow sound data. To address this issue, we will utilize contrastive learning [13, 14] to learn a pre-trained model. Contrastive learning involves training the model to distinguish between positive and negative pairs of samples, and has been shown to be effective in learning good representations even with limited data. After pre-training the model using contrastive learning, we will then fine-tune it using a smaller amount of labeled blood flow sound data from the target domain. By using contrastive learning for pre-training and fine-tuning the model with labeled blood flow sound data from the target domain, we aim to improve the performance of the deep learning model for detecting arteriovenous graft failure.

## 2 Related work

Conventional doppler ultrasonography is frequently employed for monitoring AVFs during their development following creation and if encounter any dysfunction is observed or suspected [15, 16]. Some studies had tried to evaluate and monitor the vascular access by phase-contrast magnetic resonance imaging (PC-MRI), however, it is expensive and hard to put into regular and frequent clinical application [17]. The other way is to monitor physical sign, including AVF collapse after arm elevation is lost, weak fistula, reduced arterial or increased venous pressure, limb edema, and there is less thrill in the AVF [18, 19].

On the other hand, deep learning, that involves the use of artificial neural networks, has demonstrated significant progress and adoption within the medical field. In deep learning, pretraining is a common method used to improve the performance of neural networks, especially when dealing with limited amounts of labeled data for a specific task. Pretraining involves training the model on a large dataset to learn good initial representations, which can then be fine-tuned for the specific task. In [20], the authors propose a method for pretraining audio neural networks (PANNs) using a supervised learning approach. The goal of this pretraining is to learn useful representations of audio data that can generalize to a variety of audio classification tasks. The authors use a large dataset called Audioset, which consists of over 2 million 5-second audio clips from various sources. By pretraining the PANNs on this dataset, the authors demonstrate the effectiveness of pretraining in improving the performance of neural networks for audio pattern recognition, as the PANNs achieve state-of-the-art results on several audio classification tasks.

Except for the pretrained model by a amount of labeled data, researchers have proposed various unsupervised pre-training methods, such as contrastive learning [13, 14, 21–25], which has gained popularity in the field of computer vision. In contrastive learning, the goal is to learn a model that can bring similar samples closer together and push different samples further apart, without relying on labeled data. Similar samples are pairs of data-augmented versions of the same image, called positive pairs, while different samples are pairs of data-augmented versions of different images, called negative pairs. By pre-training the model using contrastive learning, we can learn a useful initial representation that can then be fine-tuned on a smaller amount of downstream task data. This allows us to train a deep learning model even when labeled data is scarce.

In our work, we propose a pre-training approach that leverages contrastive learning with unlabeled blood flow sound data from the target domain. The goal of this pre-training is to learn robust representations that can generalize to the task of detecting graft failure. Once we obtain the pre-trained model, we fine-tune it with a limited amount of labeled blood flow sound data to derive the final graft failure detection model. The most relevant work to our study is [26]. However, the setup in [26] differs from ours. In [26], the authors either train the model from scratch or use pre-trained models with ImageNet weights. Their results indicate that the performance of ResNet with pre-trained weights is similar to that of the model trained from scratch, suggesting that conventional pre-trained image classification models may not be suitable for audio classification. In contrast, our work demonstrates that using an appropriate pre-training model, such as PANNs or models pre-trained with contrastive learning using unlabeled data, is crucial for predicting arteriovenous graft stenosis, especially when limited labeled data are available. Our approach is expected to improve the model’s performance even with limited labeled data, thereby increasing the accuracy of graft failure detection.

## 3 Data description

### 3.1 Recruitment and ethical considerations

This prospective observational study was conducted in Taiwan from September 11, 2020, to May 31, 2021. The HD patients with AVG were from the dialysis center of Kaohsiung Municipal Ta-Tung Hospital, affiliated with Kaohsiung Medical University. We excluded patients with hospital admission episodes and unstable clinical conditions. The Kaohsiung Medical University Hospital Institutional Review Board approved the study protocol. All patients provided informed written consent (KMUHIRB-E(I)-20190093).

The patients’ baseline comorbidities, clinical data, and biochemical parameters were studied. The duration of ESRD was defined as the period between the time of first dialysis and the time of enrollment in the study. The demographic features of the patients were recorded at the first visit, and the medical history was recorded using a chart review. Hypertension was defined as systolic blood pressure (BP) ≥140 mmHg, diastolic BP ≥ 90 mmHg, or the use of antihypertensive medication. Cardiovascular (CV) diseases are a clinical diagnosis of heart failure, acute or chronic ischemic heart disease, or cerebrovascular disease.

### 3.2 Data collection and labeling

Blood flow through grafts contains valuable information that can help prevent complications and improve graft longevity. The aim of this study is to develop a portable recording device that detects stenosis by extracting information from blood flow sounds. In our experiment, we used a smart electronic stethoscope named “STETH IO” to collect data. This US FDA-approved device meets stringent regulatory standards for safety and efficacy, with a 510(k) number of K160016. It belongs to the cardiovascular medical specialty, with a premarket review by the cardiac electrophysiology, diagnostics, and monitoring devices (DHT2A) division. The regulation number is 870.1875. Priced at approximately USD 90, the STETH IO offers great value for healthcare professionals seeking to improve diagnostic accuracy and patient care.

**Fig 1:**
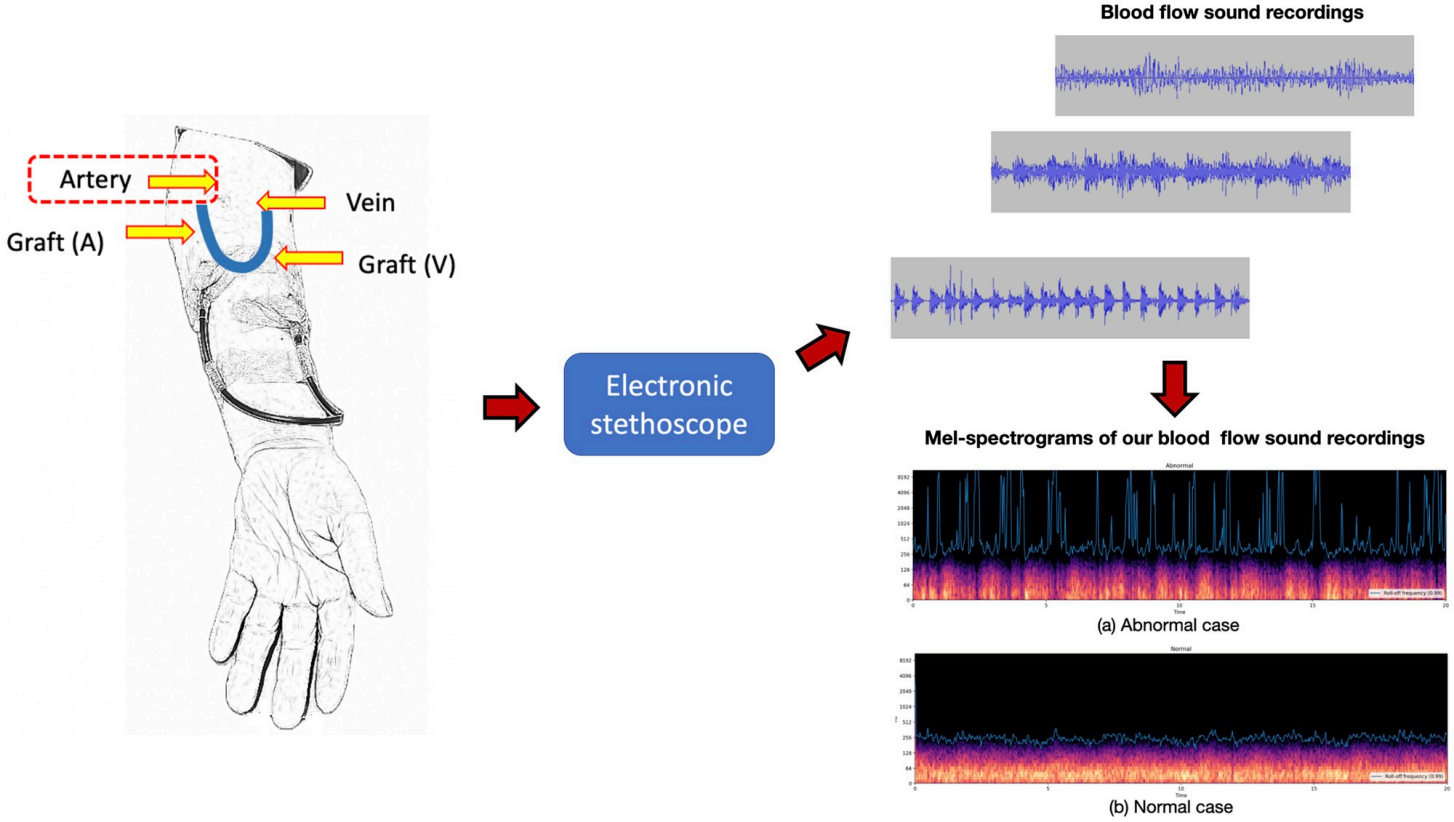
The procedure of data collecting and transformation in our work.

**Fig 2:**
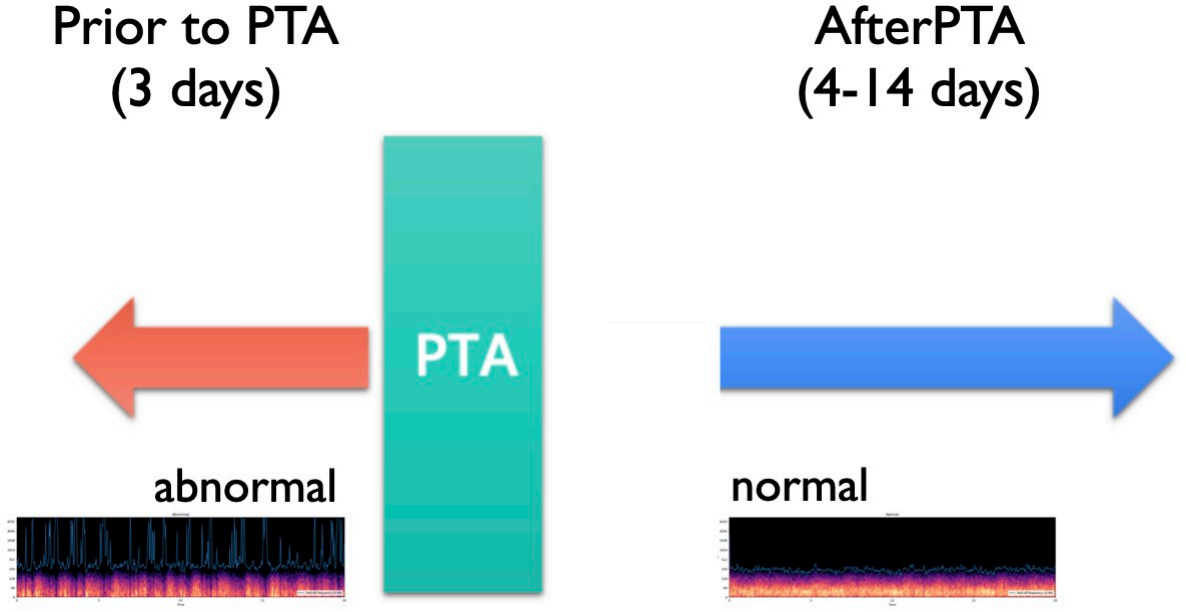
Our data labeling strategy: Recordings obtained prior to percutaneous transluminal angioplasty (PTA) were labeled abnormal and those obtained after PTA were labeled normal.

In our work, blood flow sounds were collected from arterial ends of arteriovenous fistulas on the arm as shown in Fig 1. Measurements were conducted weekly, with a one-minute recording per patient. Labels for the detection model were created according to percutaneous transluminal angioplasty (PTA) times. Recordings obtained prior to PTA were labeled abnormal and those obtained after PTA were labeled normal as shown in Fig 2. Data outside the period are unlabeled. In our work, we enrolled 45 patients with HD by AVG (Table 1). According to the selection criteria of bruit of AVG recording, majority of the locations of arteriovenous access are left arms. And in patients with AVG, there were 90% (35/39) located in forearm.

**Table 1:** Characteristics of patients ESRD.

Baseline Clinical features of the patients enrolled in the prospective study of AVS stenosis	
Patients, n	45
Baseline patient demographics and comorbidities	N (%)
Patients age, yr, mean ± SD	74 ± 10
Women	26 (57.8%)
Diabetes	26 (57.8%)
Hypertension	38 (84.4%)
Dialysis year, mean ± SD	4.6 ± 4.9
Primary Kidney Disease for dialysis	
Chronic glomerular disease	14 (31.1%)
Obstructive uropathy	3 (6.70%)
Diabetic nephropathy	27 (60.0%)
Hypertension	4 (8.90%)

In a nutshell, we have 45 patients and 676 recordings where the numbers of normal, abnormal, and unlabeled recordings are 85 and 26, and 522, respectively. In our collected data, each one-minute recording is recorded manually. To avoid the operation noise, we only extract 20 seconds in the middle of each one-minute recording.

## 4 Graft failure detection with blood flow sounds

Similar to many modern audio classification models [20, 27, 28], we transform the extracted 20-seconds recordings to mel-scaled spectrograms as Fig 1. We regard these mel-spectrograms from recordings as images and apply classic deep learning approaches in image classification, such as convolutional neural networks (CNNs), to detect the graft failure.

It is worth noting that the initial weights of deep learning models play a crucial role in determining their performance. A well-initialized model can learn faster and achieve higher accuracy than a poorly initialized one. If the initial weights are chosen poorly, the optimization may get stuck in a local minimum, resulting in suboptimal performance. On the other hand, pre-training models can provide a useful initialization for deep learning models. In pre-training, a model is first trained on a large dataset, typically with a different but related task, to learn general features that can be useful for other tasks. This pre-trained model can then be fine-tuned on a smaller dataset for the target task, with the weights initialized from the pre-training. This approach has been shown to be effective in many applications, especially when the target dataset is small, as it allows the model to leverage the knowledge learned from the larger pre-training dataset. In our work, we use different pre-training models and aim to acquire more powerful failure detection models with fine-tuning.

### 4.1 Learning model by training from scratch

By treating the mel-spectrograms as images, we were able to leverage the power of image classification models in deep learning to detect graft failure. In our work, we utilized a 14-layer convolutional neural network (CNN14) as the backbone for our approach. The CNN14 model consists of six convolution blocks and each block contain 3x3 kernel mapping, batch normalization, and rectified linear unit (ReLU) activation function as shown in Table 2. These convolution blocks are used to extract the informative visual features from the mel-spectrograms. Our choice of a relatively simple CNN architecture was motivated by the small size of the dataset and the limited complexity of the task at hand.

**Table 2:** The architecture of CNN14 in [20].

CNN14
Log-mel spectrogram (1000 frames × 64 mel bins)
{kernel: 3 × 3 @ 64 (BN, ReLU)} × 2
Max. Pooling 2x2
{kernel: 3 × 3 @ 128 (BN, ReLU)} × 2
Max. Pooling 2x2
{kernel: 3 × 3 @ 256 (BN, ReLU)} × 2
Max. Pooling 2x2
{kernel: 3 × 3 @ 512 (BN, ReLU)} × 2
Max. Pooling 2x2
{kernel: 3 × 3 @ 1024 (BN, ReLU)} × 2
Max. Pooling 2x2
{kernel: 3 × 3 @ 2048 (BN, ReLU)} × 2
Global pooling
Full Connected Layer 2048 (ReLU)
Full Connected Layer 2 (sigmoid)

**Fig 3:**
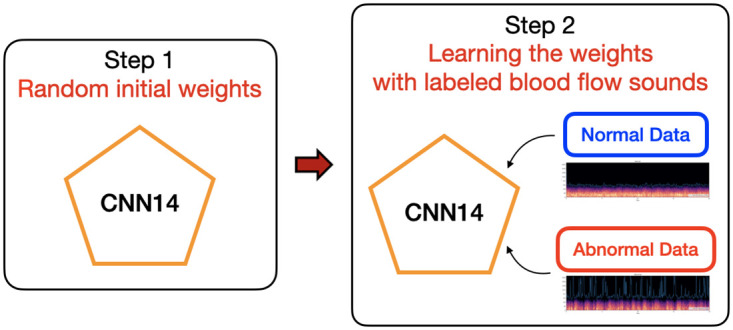
Illustration of the model train from scratch.

In our baseline model, we only used labeled data of normal and abnormal sound flow recordings for training the model from scratch as shown in Fig 3. The simple setting allowed us to effectively teach the model to distinguish between normal and abnormal recordings. It is worth noting that training from scratch is equivalent to using random initial weights for the model, which means that the performance of the model depends heavily on the quality of the data and the effectiveness of the training process.

### 4.2 Fine-tuning model with the PANNs pre-trianed model

While our CNN14 model used for graft failure detection in blood flow sounds is relatively simple, it still contains a large number of parameters. This can make training the model from scratch challenging, especially when we have limited labeled data available (90 normal and 29 abnormal recordings). In our work, we only had a few examples of labeled normal and abnormal sound flow recordings, which is not enough to effectively train a complex model. In this context, pre-training models on larger datasets for related tasks can provide a useful initialization for the target task and alleviate the issue of data scarcity. By leveraging pre-trained models, we can transfer the knowledge learned from the pre-training task to the target task and potentially achieve better performance with limited data. Therefore, pre-training models can be an effective approach for overcoming the challenges of training complex models from scratch with limited data.

**Fig 4:**
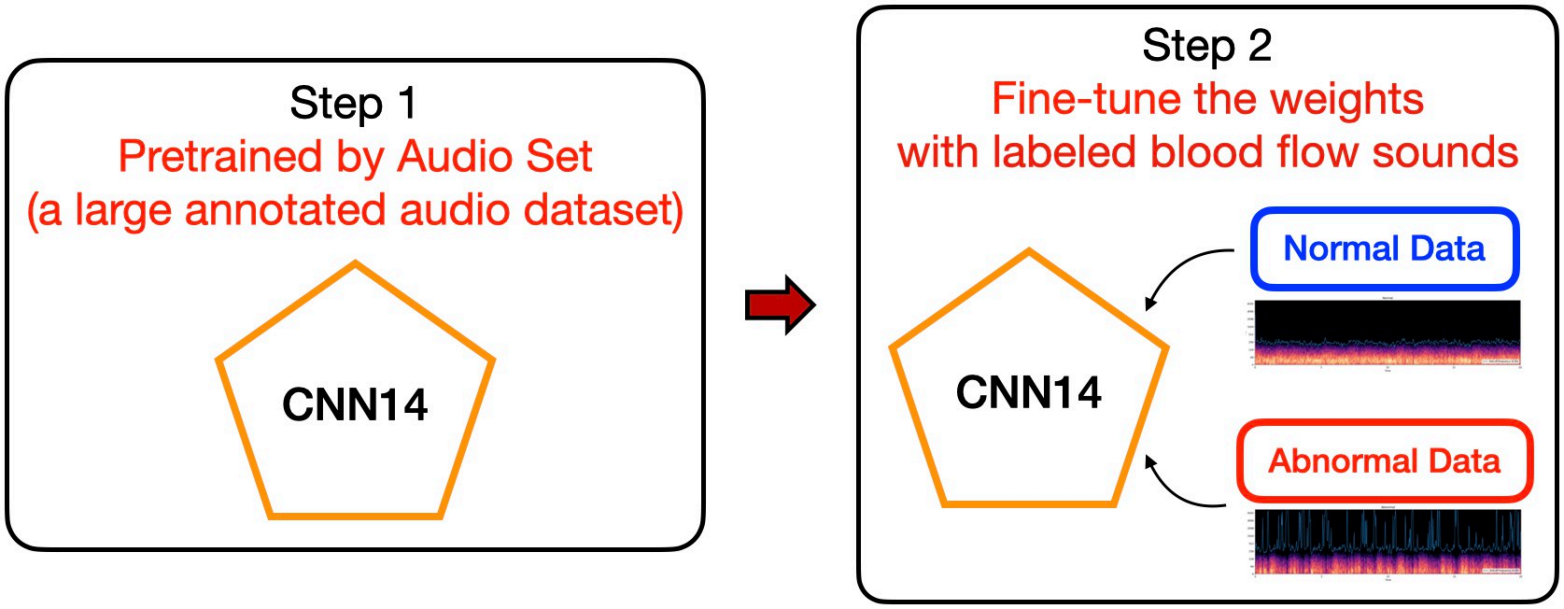
Illustration of the model fine-tuned with PANNs pre-trained model [20].

In our work, we utilized a pre-trained model called PANNs (Large-Scale Pretrained Audio Neural Networks for Audio Pattern Recognition [20]) for graft failure detection in blood flow sounds. PANNs is a pre-trained model that was trained on the large-scale AudioSet dataset, which includes a diverse set of labeled audio samples covering a wide range of audio events. The model is built on a CNN14 backbone architecture and was fine-tuned using our labeled sound flow data to adapt it to our specific task of graft failure detection. By fine-tuning the pre-trained model, which provides an initial set of weights from a related domain as shown in Fig 4, we were able to transfer the knowledge learned from the large AudioSet dataset to our smaller dataset and effectively learn the relevant features for our target task. This approach enabled us to develop an accurate and reliable model for detecting graft failure in blood flow sounds, without the need for training the model from scratch.

### 4.3 Improving pre-training model by contrastive learning

While pre-training models can provide an initial weight from a related domain, it can also suffer from biases that may not be optimized for a specific task. To address this issue, we leveraged the availability of unlabeled data by applying self-supervised learning (SSL) [13, 21, 29] for pre-training the CNN14 model. Specifically, we utilized a contrastive learning approach, which learns representations by maximizing the similarity between augmented versions of the same sample and minimizing the similarity between augmented versions of different samples. This allowed us to utilize the 557 unlabeled data to pre-train the CNN14 model and improve its performance. By using self-supervised learning, we were able to overcome the limitations of pre-training on a large-scale dataset, and tailor the model to the specific characteristics of our task.

#### Contrastive learning

**Fig 5:**
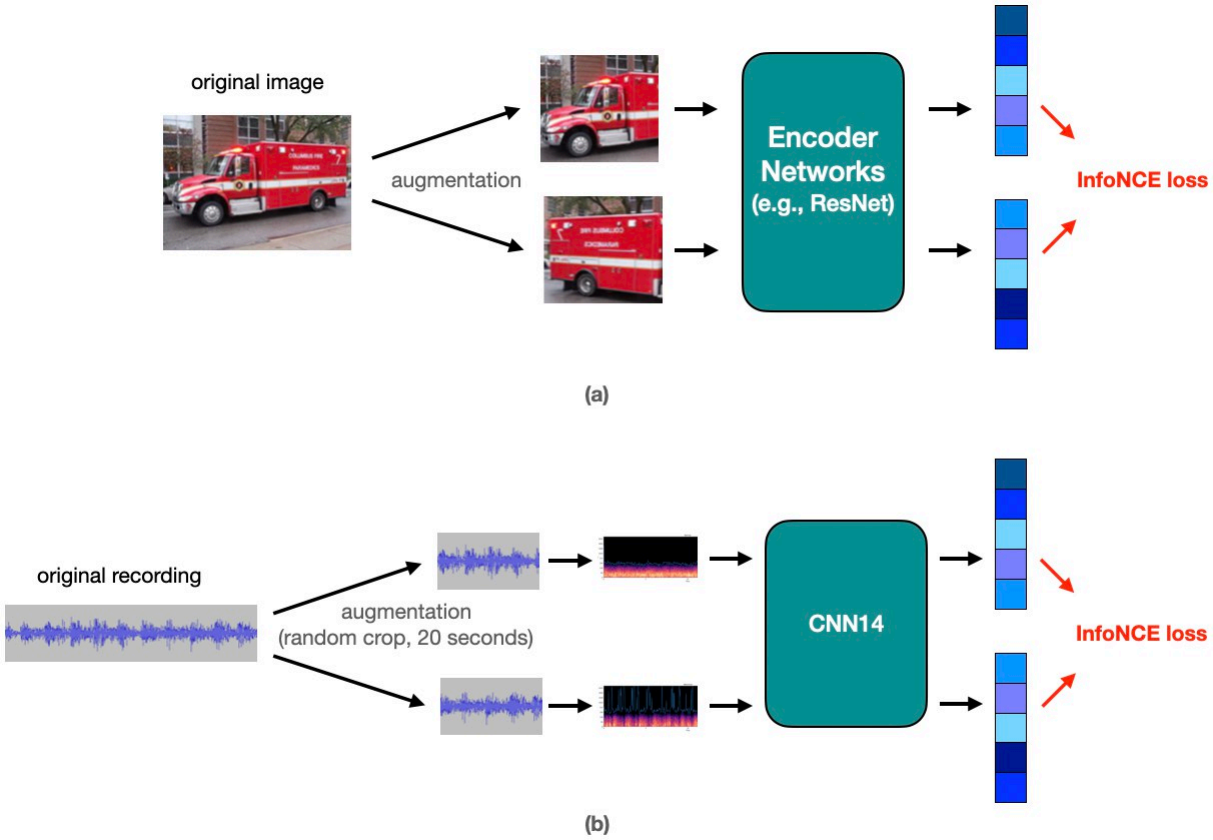
(a)Illustration of contrastive learning with image data (b)The contrastive learning with our unlabeled blood flow sounds in our work.

Many SSL approaches have been proposed to learn pre-trained models with self-supervision. One of these popular SSL approaches is the constrative learning [13, 14, 21, 22, 25]. Constrative learning aims to learn an embedding space (or representation) in which similar sample pairs stay close to each other while dissimilar ones are far apart. As an example, in image classification, we can use contrastive learning to learn representations that can differentiate between different images. More specifically, we can take two images, one as an anchor image and the other as a positive or negative image, depending on whether the two images belong to the same class or not. The model learns to map both images to a common feature space such that the representations of the anchor and positive images are pulled closer together, while the representations of the anchor and negative images are pushed further apart as shown in Fig 5(a).

In contrastive learning, the infoNCE loss is used to encourage the model to learn to differentiate between similar (positive) and dissimilar (negative) pairs of images where NCE stands for Noise-Contrastive Estimation [30]. Assume we random sample a minbatch of *N* images. Each image can be augmented by the random crop to produce similar pairs (*z*_*i*_ and *z*_*j*_), resulting in 2*N* data samples. The infoNCE loss L of the contrative learning is defined as follows:

$$\begin{array}{c} L=\frac{1}{2N}\sum_{k=1}^{N}\left[\ell \left(2k-1,2k\right)+\ell \left(2k,2k-1\right)\right], \end{array}$$ (1)

where

$$\begin{array}{c} \ell \left(i,j\right)=-\text{log}\frac{\text{exp}(\text{sim}\left(z_{i},z_{j}\right)/\tau )}{\sum_{k=1}^{2N}1_{k\neq i}\text{exp}\left(\text{sim}\left(z_{i},z_{k}\right)/\tau \right)} \end{array}$$ (2)

is the loss of similar pairs and *sim*(*u*, *v*) = *u*^⊤^*v*/‖*u*‖‖*v*‖ and *τ* denotes a temperature parameter. The model is trained using stochastic gradient descent to minimize the infoNCE loss.

#### Contrastive learning with our unlabeled blood flow sounds

In contrast to using pre-trained models such as PANNs, we utilized contrastive learning to pre-train our model using our own dataset of interest. Specifically, we had a set of 557 unlabeled sound data that we used for pre-training. In our contrastive learning approach as shown in Fig 5(b), we augmented the data through random crops (20 seconds) of the recordings, rather than using mel-spectrograms. This strategy of data augmentation allowed for stronger data augmentation and better representation learning, which is crucial for detecting subtle differences in blood flow sounds indicative of graft failure.

**Fig 6:**
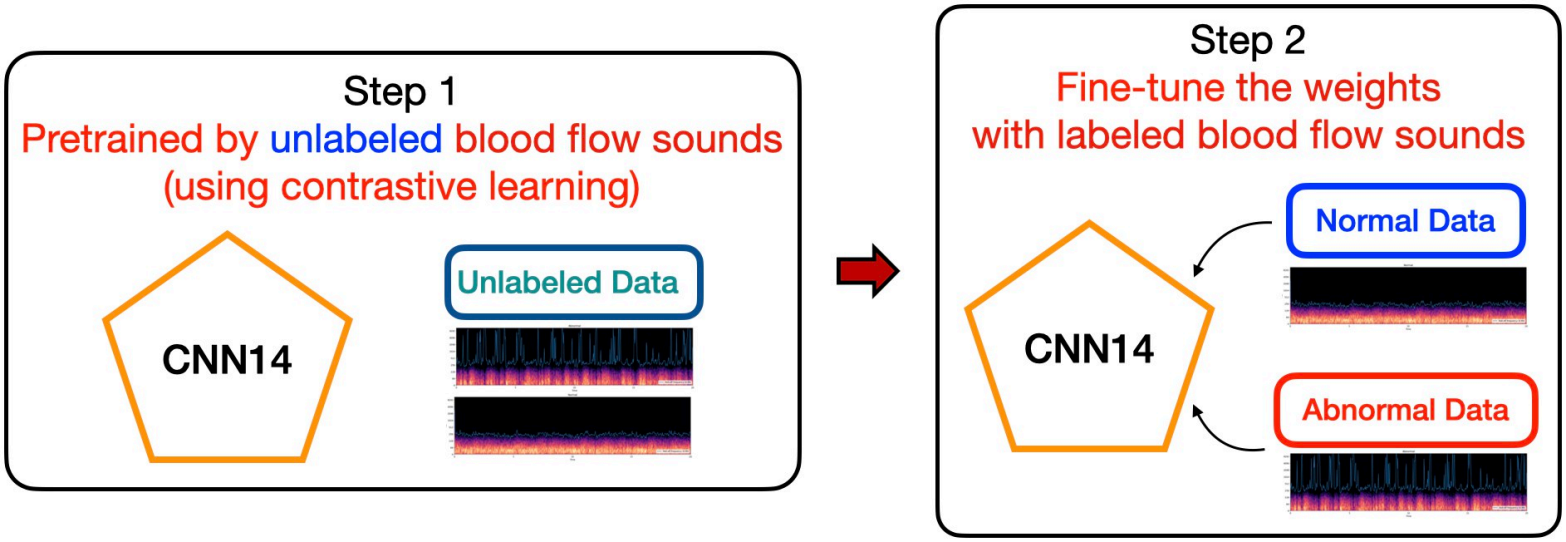
Our pre-trained model by contrastive learning with unlabeld blood flow sounds.

After pre-training our model using contrastive learning with our own dataset, we obtained initial weights that were tailored specifically to our task. We then further fine-tuned the pre-trained CNN14 model using labeled normal and abnormal blood flow sound data as shown in Fig 6. This fine-tuning step allowed us to improve the model’s performance on our specific classification task by adjusting the weights to better fit the labeled data. This approach of first pre-training with unlabeled data and then fine-tuning with labeled data has been shown to be effective in our experiments and has the advantage of being able to utilize both labeled and unlabeled data for model training.

## 5 Experiment

### 5.1 Setting and implementation detail

For our experiments, we utilized a 5-fold cross-validation approach. Each fold contained a similar proportion of samples indicating graft failure and those that did not. Specifically, 4 folds were used for training the model, while the remaining fold was used for validation and testing. Within this fold, we employed a leave-one-out testing approach. This allowed us to thoroughly evaluate the model’s performance on unseen data and ensure that the results were robust and not specific to a particular subset of the data.

Our experiments were conducted using the CNN14 backbone model. We conducted three different training settings, which included training the model from scratch for 40 epochs with a batch size of 16 and a learning rate of 0.0001. In the second setting, we fine-tuned the model using a PANNs pre-trained model for 20 epochs, using the same hyperparameters as the first setting. Finally, we utilized contrastive learning, training the model for 200 epochs and evaluating the status of the pretraining model every 10 epochs. During the contrastive learning training, we used a batch size of 64 and a learning rate of 0.0001, with the Adam optimizer applied in all settings. Within each batch, every recording is randomly truncated into two 5-second segments as part of the data augmentation process. Once the pre-trained model is obtained, we fine-tune it using the labeled data from the four training folds. It is worth noting that data augmentation is not applied during the fine-tuning and testing phases.

### 5.2 Results

We evaluated the performance of three different methods to predict the failure of arteriovenous grafts (AVG) using deep learning algorithms. The first method involved training the model from scratch, the second method used a pre-trained model based on PANNs, and the third method used pre-training via contrastive learning. Additionally, we included a method where professional nephrology nurses classified the blood flow sounds by listening to the recordings. Two human evaluators, both nephrology nurses, participated in this evaluation.

In our experiment, we used several measurements to evaluate the performance of our proposed model. These measurements include accuracy, precision, recall, F1 score, and AUC (area under the curve). The measurements commonly used to evaluate classification models and the formulations are:

$$\begin{array}{c} Accuracy=\frac{TP+TN}{TP+TN+FP+FN}, \end{array}$$

$$\begin{array}{c} Precision=\frac{TP}{TP+FP}, \end{array}$$

$$\begin{array}{c} Recall=\frac{TP}{TP+FN}, \end{array}$$

$$\begin{array}{c} F1=2\cdot \frac{Precision\cdot Recall}{Precision+Recall}, \end{array}$$

and

$$\begin{array}{c} AUC:integration\;of\;the\;ROC\;curve \end{array}$$

where where TP represents true positives, TN represents true negatives, FP represents false positives, and FN represents false negatives.

**Fig 7:**
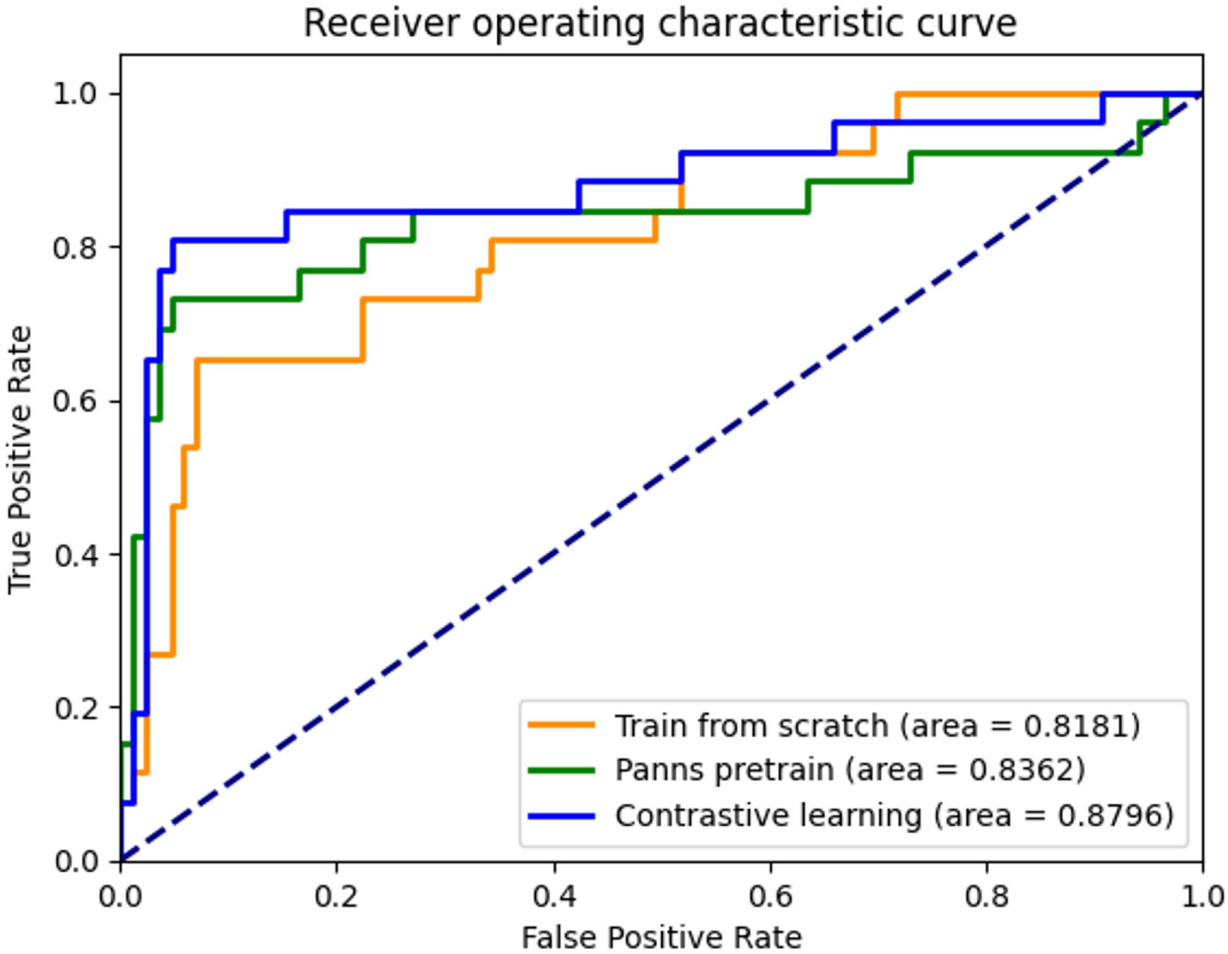
ROC curves of of the model training from scratch, fine-tuning model with PANNs, and fine-tuning model with pre-training via contrastive learning.

Based on the results of Tabel 3 and Fig 7, it can be inferred that the PANNS pre-trained model outperformed the model trained from scratch. The former achieved an accuracy of 0.8919, higher than the latter’s accuracy of 0.8649. Similarly, the PANNS pre-trained model achieved higher precision (0.8182 vs. 0.7391), recall (0.6923 vs. 0.6538), and F1-score (0.7500 vs. 0.6939) compared to the model trained from scratch. It is noteworthy that the PANNS pre-trained model leverages proper initial weights geared from a pre-training process on a larger dataset in a different domain. Such an approach could enable the model to acquire generic features that are transferable to the downstream task, thereby enhancing its performance. Additionally, the proper initialization of the model’s weights using pre-trained weights allows it to start the training process with a better understanding of the features and relationships present in the data, which could lead to better convergence and performance. The results also shows practical implications for improving the performance of detection models, especially in scenarios where data may be limited, and pre-trained models could provide a viable alternative.

**Table 3:** Accuracies, precisions, recalls, F1 scores, and AUCs of the model trained from scratch, fine-tuned with PANNs, fine-tuned with pre-training via contrastive learning, and evaluated by two professional nephrology nurses.

Method	Accuracy	Precision	Recall	F1	AUC
Tram from scratch	0.8649	0.7391	0.6538	0.6939	0.8181
PANNs pre-training	0.8919	0.8182	0.6923	0.7500	0.8362
Contrative learning	**0.9279**	**0.8462**	**0.8077**	**0.8400**	**0.8796**
Human evaluator 1	0.7117	0.4400	0.8461	0.5789	-
Human evaluator 2	0.7837	0.5294	0.6923	0.5999	-

The comparison between the PANNs pre-trained model and the model pre-trained via contrastive learning with unlabeled target domain data (unlabeled blood flow sounds) indicates that the latter outperforms the former in all performance metrics, as shown in Table 3. The model pre-trained via contrastive learning achieved higher accuracy, precision, recall, F1-score, and AUC value. This improvement can be attributed to the utilization of additional unlabeled target domain data, which enhances the model’s feature representation. The results highlight the importance of using data from the target domain during the pre-training process to improve the model’s performance in that specific domain.

In our final comparison, we included an evaluation by two professional nephrology nurses who classified the blood flow sounds by listening to the recordings. One nurse achieved an accuracy of 0.7117, recall of 0.8461, precision of 0.4400, and an F1 score of 0.5789. The other nurse achieved an accuracy of 0.7837, recall of 0.6923, precision of 0.5294, and an F1 score of 0.5999. These results, shown in Table 3, demonstrate the effectiveness of our proposed framework and highlight the challenge of relying solely on blood flow sounds for accurate classification. Furthermore, our results suggest that pre-training with unlabeled target domain data is a more effective approach compared to training from scratch or using pre-trained models from other domains. This pre-training approach is especially beneficial when labeled data is limited or expensive to obtain. The ease of collecting unlabeled data from the target domain also makes this pre-training approach a cost-effective and efficient alternative.

## 6 Discussion

### 6.1 Summary of main findings

Our study demonstrates the feasibility of converting audio signals into Mel-spectrograms and subsequently leveraging CNNs to autonomously discern salient features, thus obviating the necessity for manual feature extraction rooted in human domain expertise. This methodology holds promise in scenarios where the pertinent features are not readily identifiable a priori or where the manual extraction of features poses significant challenges due to complexity or resource intensiveness. The proposed approach underscores the potential for automated systems to effectively uncover intricate patterns in audio data, offering a robust solution for various applications in which feature extraction presents a formidable bottleneck.

Our study also underscores the potential efficacy of SSL techniques in clinical applications, particularly in contexts characterized by limited availability of labeled data. Specifically, we employed contrastive learning to facilitate pre-training of a model using unlabeled data, subsequently fine-tuning it for a specific downstream task. Our findings indicate that pre-training on relevant unlabeled data significantly enhances the model’s performance on the downstream task, surpassing the outcomes achieved from scratch, even when labeled data is scarce. This observation demonstrates the utility of SSL as a viable strategy for enhancing the performance of machine learning models in clinical applications, where the acquisition of labeled data is often constrained by cost and availability. Our results suggest that pre-training with unlabeled target domain data is more practical than training from scratch or using pre-training models from other domains. This pre-training approach is especially beneficial when labeled data is limited or expensive. The ease of collecting unlabeled data from the target domain also makes this pre-training approach a cost-effective and efficient alternative.

This study investigated optimal data collection methodologies for blood flow sound analysis in vascular access monitoring, highlighting the superiority of arterial end recordings over graft arterial recordings. Furthermore, we emphasized the pivotal role of proper data labeling in enhancing model learning outcomes. Specifically, our study implemented a labeling strategy based on PTA times, designating recordings acquired before PTA as abnormal and those obtained four days post-PTA as usual. These findings underline the significance of careful selection of data collection approaches and labeling techniques, which can significantly impact the quality and utility of data for machine learning applications in clinical settings.

### 6.2 Clinical implications

The primary patency rates of AVG at 12, 24, and 36 months were found to be significantly low, at 20.4%, 7.4%, and 5.0%, respectively. The known pathological reasons that lead to the insufficient long-term use of AVG include fluid-wall shear stress, vascular cells (including endothelial cells, smooth muscle cells, etc.) lesions, and other effects. In AVG stenosis, a large amount of arterial blood that violates the normal physiological function directly enters the vein, which changes the hemodynamics of the vein. Coupled with the generation of stenotic obstruction, perturbed flow will lead to more complex blood flow and activation of various transduction pathways, leading to a vicious circle of upregulation of pro-proliferative and pro-thrombotic expression genes [31–33]. Complex blood flow often exists in areas of stenosis or stenosis of vessel bifurcations, and these sites increase the presence of atherosclerosis in neointimal hyperplasia [34, 35].

Our study introduces a novel approach by utilizing noninvasive and easily accessible blood flow sounds recorded, as opposed to sonography accessed by healthcare staff, to predict early AVG failure. Moreover, our well-initialized model with fine-tuning, based on contrastive learning, can be personalized for future clinical applications, requiring a smaller dataset for IoT integration into an early-warning system. This has significant implications for improving patient care and outcomes by facilitating timely interventions to prevent AVG failure in hemodialysis patients.

### 6.3 Limitations and future directions

Our work has several limitations that should be considered when interpreting the results:

- Determining the appropriate period for labeling the data based on PTA relied on domain knowledge, which might introduce subjectivity and variability. Further research is needed to establish a more objective and standardized approach to labeling.
- Our model might be sensitive to background noise due to the limited labeled data available for training. More labeled data is needed to improve the robustness of the model to noise.
- The need for interpretability of the model is a common challenge in machine learning, and further research is required to develop methods for explaining the model’s predictions. Overall, these limitations highlight the need for continued research to improve the reliability and utility of machine-learning models in clinical applications.

## 7 Conclusion

We investigated the effect of applying convolutional neural networks to AVG’s blood flow sound for early stenosis prediction. Unlike previous methods that rely on analyzing AVG stenosis in angiography and staff experience to determine malfunction significance, our proposed method only analyzes AVG blood flow sound. It encodes it to classify patients into those with and without functionally significant AVG. We leveraged contrastive learning with unlabeled data to improve our model’s performance, which showed promising results. Our proposed framework demonstrated its suitability for predicting AVG failure by analyzing AVG bruit alone, offering a noninvasive and more cost-effective approach.
